# Cdk1 orders mitotic events through coordination of a chromosome-associated phosphatase switch

**DOI:** 10.1038/ncomms10215

**Published:** 2015-12-17

**Authors:** Junbin Qian, Monique Beullens, Jin Huang, Sofie De Munter, Bart Lesage, Mathieu Bollen

**Affiliations:** 1 Laboratory of Biosignaling & Therapeutics, Department of Cellular and Molecular Medicine, University of Leuven, Campus Gasthuisberg, O&N1, Herestraat 49, Box 901, Leuven B-3000, Belgium; 2 Department of Biochemistry and Molecular Biology, Guizhou Medical University, Guiyang, Guizhou 550025, China

## Abstract

RepoMan is a scaffold for signalling by mitotic phosphatases at the chromosomes. During (pro)metaphase, RepoMan-associated protein phosphatases PP1 and PP2A-B56 regulate the chromosome targeting of Aurora-B kinase and RepoMan, respectively. Here we show that this task division is critically dependent on the phosphorylation of RepoMan by protein kinase Cyclin-dependent kinase 1 (Cdk1), which reduces the binding of PP1 but facilitates the recruitment of PP2A-B56. The inactivation of Cdk1 in early anaphase reverses this phosphatase switch, resulting in the accumulation of PP1-RepoMan to a level that is sufficient to catalyse its own chromosome targeting in a PP2A-independent and irreversible manner. Bulk-targeted PP1-RepoMan also inactivates Aurora B and initiates nuclear-envelope reassembly through dephosphorylation-mediated recruitment of Importin β. Bypassing the Cdk1 regulation of PP1-RepoMan causes the premature dephosphorylation of its mitotic-exit substrates in prometaphase. Hence, the regulation of RepoMan-associated phosphatases by Cdk1 is essential for the timely dephosphorylation of their mitotic substrates.

## Abstract (web summary)

RepoMan is a signalling scaffold for mitotic phosphatases PP1 and PP2A-B56, which regulate targeting of Aurora B and RepoMan respectively, to the chromosomes. Here Qian *et al.* show that Cdk1 phosphorylates RepoMan to modulate the binding of PP1 and PP2A-B56, contributing to orderly mitotic progression.

## Graphical abstract

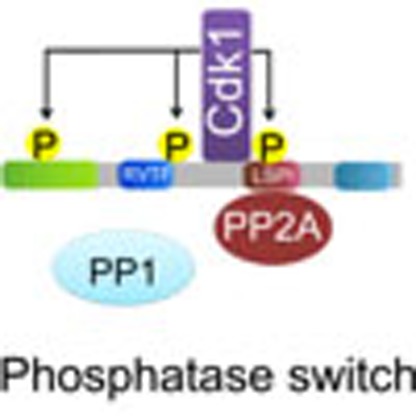

Cyclin-dependent kinase 1 (Cdk1) is the master regulator of mitosis^1^. It is activated at the G2-M transition, reaches its maximal activity in prometaphase and is inactivated at the metaphase–anaphase transition. Cdk1 triggers the mitotic entry and controls the initial phases of mitosis by phosphorylation of key regulators of processes as diverse as chromosome condensation, spindle assembly and nuclear-envelope breakdown. In addition, Cdk1 restrains proteins that are only needed at later stages of mitosis. Among the most important Cdk1 substrates are other mitotic kinases, including Aurora B and Haspin^2^,^3^,^4^. However, Cdk1 also regulates protein phosphatases PP1 and PP2A, the main mitotic phosphatases in vertebrates^5^,^6^. PP1 forms heterodimers or heterotrimers with a host of regulatory subunits that define when and where the phosphatase acts. Cdk1 inhibits PP1 both by preventing the binding of some regulatory subunits and by decreasing the specific activity of the catalytic subunit^7^,^8^. PP2A holoenzymes usually consist of a catalytic subunit, a scaffolding A subunit and a variable, substrate-specifying B subunit. The mitotically important B subunits are isoforms of B55 and B56. Cdk1 inhibits PP2A-B55 and a kinetochore pool of PP2A-B56 by activation of inhibitory proteins^9^,^10^. In contrast, Cdk1 promotes the recruitment of PP2A-B56 to the kinetochore protein BubR1 (ref. 11).

The phosphatase scaffold RepoMan, also known as CDCA2, binds directly to both PP1 (ref. 12) and PP2A-B56 (refs 13, 14). In prometaphase, RepoMan-associated PP1 dephosphorylates Histone H3 at Thr3 (H3T3) on the chromosome arms^15^. Since phosphorylated (ph) H3T3 serves as a docking site for Survivin, a subunit of the Aurora-B or chromosomal passenger complex (CPC), the selective dephosphorylation of H3T3ph on the chromosome arms contributes to the centromeric enrichment of the CPC^16^,^17^,^18^. Centromeric H3T3ph is protected from dephosphorylation by PP1-RepoMan until anaphase because RepoMan is prevented from binding to histones through Aurora-B-mediated phosphorylation at S893, in particular at the centromeres where Aurora B is most active^13^. The centromeric targeting of the CPC is further enhanced by a reciprocal positive feedback loop between Aurora B and Haspin, the H3T3 kinase^19^. Importantly, the chromosome targeting of RepoMan during (pro)metaphase is highly dynamic, because RepoMan-associated PP2A-B56 counteracts Aurora B by dephosphorylating RepoMan at S893 (ref. 13). Thus, the interplay between Haspin, Aurora B and RepoMan-associated phosphatases couples reciprocal positive and double-negative feedback loops to establish a robust and dynamic mechanism for the centromeric targeting of the CPC in (pro)metaphase (Supplementary Fig. 1)^6^. At the beginning of anaphase, the CPC is translocated to the spindle midzone enabling the complete dephosphorylation of RepoMan at S893 and its bulk recruitment to chromosomes. Considerable evidence indicates that the RepoMan-associated phosphatase activity then catalyses the dephosphorylation of hitherto unidentified substrates that contribute to chromosome segregation, chromosome decondensation and nuclear-envelope reassembly^8^,^20^,^21^.

Cdk1 promotes the centromeric targeting of the CPC at different levels (Supplementary Fig. 1). It phosphorylates and activates Haspin^3^,^4^, but also enhances CPC binding to centromeric Shugoshin through phosphorylation of the CPC subunit Borealin^2^. In addition, Cdk1 opposes the chromosomal targeting of RepoMan^8^,^20^, but the underlying mechanism and the importance of this regulation are not clear. Here, we show that Cdk1 has opposite effects on RepoMan-associated PP1 and PP2A-B56. Cdk1 reduces the binding and activity of PP1 to levels that are sufficient to keep the chromosome arms dephosphorylated at H3T3, but below the threshold needed to dephosphorylate centromeric H3T3 and other anaphase substrates. On the other hand, Cdk1 promotes the recruitment of PP2A-B56, which dephosphorylates RepoMan at S893 and thereby balances Aurora B to set the level of chromosome-targeting of RepoMan in (pro)metaphase. The inactivation of Cdk1 at the beginning of anaphase induces a phosphatase switch on RepoMan that culminates in the bulk recruitment of PP1-RepoMan to chromosomes and the timely dephosphorylation of key regulators of the mitotic exit.

## Results

### Cdk1 limits the chromosome targeting of RepoMan

**Figure 1:**
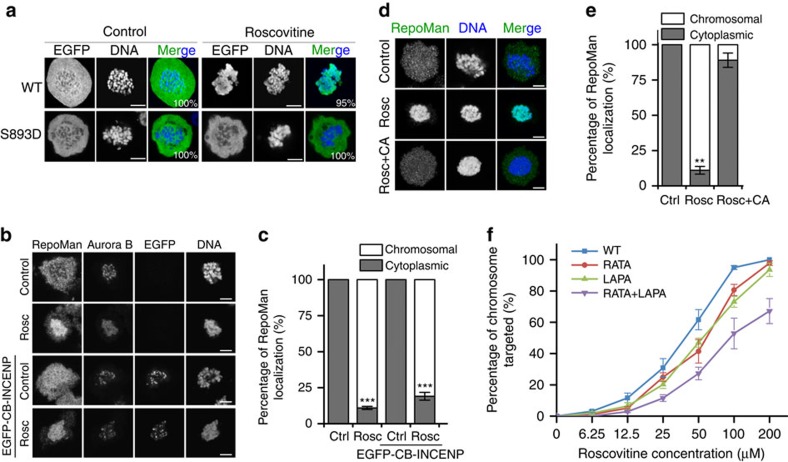
Cdk1 opposes the chromosome targeting of RepoMan. (**a**) Nocodazole-arrested U2OS cells transiently expressing EGFP-tagged RepoMan-WT or RepoMan-S893D were treated with dimethyl sulfoxide (DMSO) (control) or Roscovitine (100 μM) for 30 min before fixation and visualization of EGFP and DNA. The numbers in the figures refer to the percentage of cells where the shown phenotype occurred. Scale bars, 5 μm. (**b**) Confocal images of Nocodazole-arrested U2OS cells, in the absence or presence of Roscovitine (Rosc) and transiently expressed EGFP-tagged CENP-B (CB) fused to INCENP. The fixed cells were stained for endogenous RepoMan, Aurora B and DNA. Scale bars, 5 μm. (**c**) Quantification of RepoMan localization in **b**. The graphs show mean percentages±s.e.m. from three independent experiments (≥73 cells for each condition per experiment). ***P<0.001 in paired *t*-test, compared with control (Ctrl). (**d**) Nocodazole-arrested U2OS cells were treated with DMSO (control) or 100 μM Roscovitine for 30 min, as such or in combination with 20 nM Calyculin A (CA). The fixed cells were stained for endogenous RepoMan and DNA. Scale bars, 5 μm. (**e**) Quantification of EGFP-RepoMan localization in **d**. Graphs show mean percentages±s.e.m. from three independent experiments (≥74 cells for each condition per experiment). **P<0.01 in paired *t*-test, compared with control (Ctrl). (**f**) Nocodazole-arrested U2OS cells expressing the indicated EGFP-tagged RepoMan variants were incubated for 30 min with different concentrations of Roscovitine before fixation. DNA was stained with DAPI. The figure shows the percentage of cells where the EGFP-fusion was chromosome-associated. The results are expressed as means±s.e.m. from three independent experiments (≥103 cells per data point).

The inhibition of Cdk1 induces the recruitment of RepoMan to chromosomes in chicken DT40 cells^8^,^20^. We noted that the Cdk1 inhibitor Roscovitine (Fig. 1a) also induced a massive chromosome targeting of ectopically expressed EGFP-tagged RepoMan in prometaphase-arrested U2OS cells. This effect was not seen with the S893D mutant. Since S893 phosphorylation or the S893D mutation preclude the binding of RepoMan to histones^13^, these findings suggested that Cdk1 inhibition caused the chromosome targeting of RepoMan through a reduced activity of Aurora B and/or the activation of a counteracting phosphatase. The (auto)activation of Aurora B is critically dependent on its centromeric enrichment^22^ and Cdk1 enhances centromeric CPC recruitment through both the Borealin-Shugoshin and Survivin-H3T3ph pathways^2^,^16^,^17^,^18^. Accordingly, Roscovitine caused the loss of Aurora B from the centromeres (Fig. 1b). However, the artificial targeting of Aurora B to centromeres by overexpression of the CPC component INCENP fused to the centromeric protein CENP-B^19^ did not significantly reduce the Roscovitine-induced chromosome hypertargeting of RepoMan (Fig. 1b,c). In contrast, this Roscovitine effect was largely abolished by Calyculin A (Fig. 1d,e), an inhibitor of PP1 and PP2A(-like) phosphatases, suggesting a dependency on phosphatase activity. Since both PP1 and PP2A-B56 are associated with RepoMan during prometaphase, we subsequently examined whether Roscovitine had distinct effects on the chromosome targeting of previously characterized phosphatase-binding mutants of RepoMan^13^. Dose–response curves revealed a reduced sensitivity to Roscovitine after mutation of either the RVxF-type PP1-binding site (RATA mutant) or the LSPI-type B56-binding site (LAPA mutant) (Fig. 1f). The combined mutations had an additive effect. This suggested that the Roscovitine-induced chromosome hypertargeting of RepoMan was dependent on associated PP1 and PP2A.

### Cdk1 has opposite effects on the binding of PP1 and PP2A-B56

**Figure 2:**
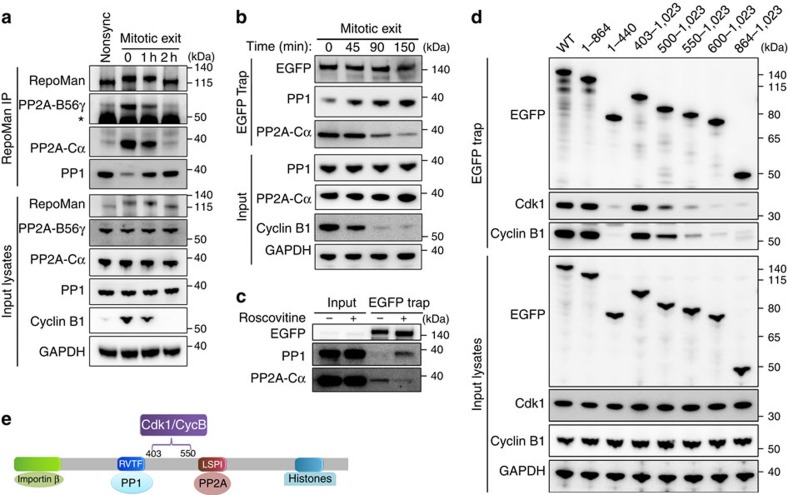
Cdk1 regulates the association of PP1 and PP2A-B56 with RepoMan. (**a**) Nocodazole-arrested prometaphase U2OS cells were collected by shake-off and then released into fresh medium for different time points to synchronize the cells for mitotic exit. Immunoprecipitates (IP) of endogenous RepoMan were prepared from lysates of these synchronized cells as well as from non-synchronized cells. The IPs were analysed by immunoblotting with the indicated antibodies. Asterisk denotes IgG-heavy chain. (**b**) U2OS cells were transfected with EGFP-RepoMan-WT and arrested/released as in **a**. EGFP traps of cell lysates were analysed by immunoblotting. (**c**) Nocodazole-arrested U2OS cells were collected by shake-off and then treated with or without 100 μM Roscovitine for 30 min. EGFP traps from cell lysates were analysed by immunoblotting. (**d**) EGFP traps of lysates from Nocodazole-arrested HEK293T cells expressing EGFP fusions of the indicated RepoMan fragments were processed for immunoblotting with the indicated antibodies. (**e**) Map of the protein-binding sites of RepoMan.

To understand how Cdk1 regulates the RepoMan-associated phosphatases, we first examined how the binding of PP1 and PP2A-B56 to endogenous RepoMan evolved after the release of U2OS cells from a Nocodazole arrest in prometaphase (Fig. 2a). During prometaphase, little PP1 was associated with immunoprecipitated RepoMan, but the binding of PP1 increased considerably during anaphase when the Cdk1-regulatory subunit Cyclin B got degraded. Eventually, the association of PP1 with RepoMan at the end of mitotic exit was similar to that in non-synchronized cells. In contrast, the binding of PP2A-B56 to RepoMan was maximal in prometaphase, rapidly decreased in anaphase and was nearly undetectable 2 h after the Nocodazole release and in non-synchronized cells. Similar results were obtained for ectopically expressed EGFP-tagged RepoMan (Fig. 2b). Moreover, a shift from PP2A to PP1 binding could also be provoked by the inhibition of Cdk1 with Roscovitine (Fig. 2c), indicating that this phosphatase switch is controlled by Cdk1. This notion was consistent with the finding that both Cdk1 and Cyclin B co-precipitated with EGFP-RepoMan (Fig. 2d). Using deletion mutagenesis the Cyclin B/Cdk1-binding site was mapped to residues 403–550, between the binding sites of PP1 and PP2A-B56 (Fig. 2e).

### Cdk1 opposes PP1-RepoMan

**Figure 3:**
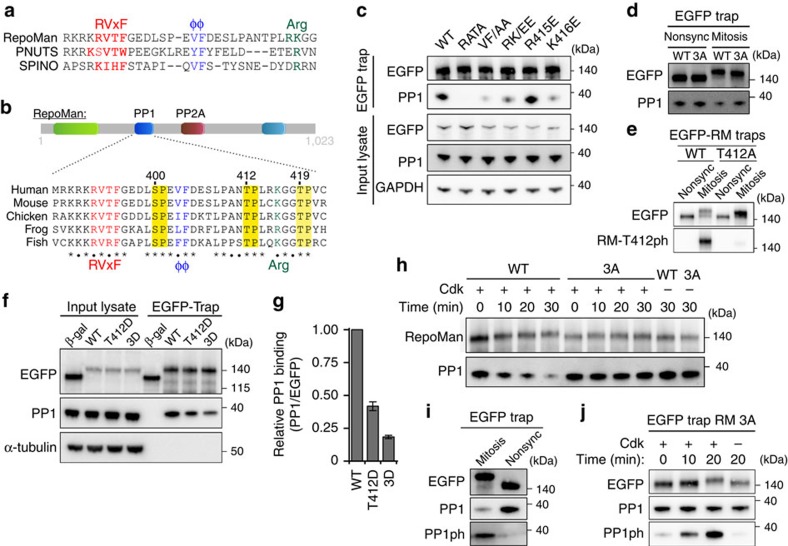
Cdk1 reduces the activity of PP1-RepoMan. (**a**) Sequence alignment of the PP1-interaction domains of RepoMan, PNUTS and Spinophilin. (**b**) Conservation of the PP1-interaction domain of RepoMan in vertebrates. (**c**) EGFP traps from HEK293T cells expressing the indicated EGFP-RepoMan mutants were analysed by immunoblotting for the binding of PP1. (**d**) U2OS cells were transfected with EGFP-tagged RepoMan-WT or RepoMan-3A. EGFP traps from non-synchronized (Nonsync) or Nocodazole-arrested (Mitosis) U2OS cell lysates were examined for associated PP1. (**e**) U2OS cells were transfected with EGFP-tagged RepoMan-WT. EGFP traps from Nonsync or Nocodazole-arrested (Mitosis) U2OS cell lysates were examined for EGFP and RepoMan-T412ph antibody. (**f**) EGFP traps from non-synchronized HEK293T cells expressing EGFP-tagged β-galactosidase (β-gal), RepoMan-WT, RepoMan-T412D or RepoMan-3D were analysed by immunoblotting for associated PP1. (**g**) The PP1/EGFP ratio was quantified by densitometric analysis. The data represent means±s.e.m. from three independent experiments. (**h**) EGFP traps from non-synchronized HEK293T cells expressing EGFP-tagged RepoMan-WT or RepoMan-3A were phosphorylated *in vitro* with recombinant Cdk2/Cyclin A. Unbound PP1 was washed away before the addition of SDS sample buffer to the traps and immunoblotting for PP1. (**i**) Phosphorylation of the C-terminal Cdk site of PP1 (PP1ph) was examined by immunoblotting of EGFP traps from U2OS cells expressing EGFP-RepoMan-WT in Nocodazole-arrested (Mitosis) or non-synchronized (Nonsync) U2OS cells with a phospho-epitope specific antibody. (**j**) EGFP traps from non-synchronized HEK293T cells expressing EGFP-RepoMan-3A (RM 3A) were phosphorylated with Cdk2/Cyclin A. The unbound fractions were washed away before the addition of SDS sample buffer to the traps and immunoblotting for PP1 and PP1ph.

To find out how Cdk1 affects PP1-RepoMan, we first performed an *in silico* analysis of the PP1-binding domain of RepoMan. Recent structural data revealed that regulatory subunits of PP1 often have multiple PP1-binding motifs^23^. For example, PNUTS and Spinophilin contain a tripartite PP1-binding domain that comprises appropriately spaced RVxF, ΦΦ and Arg binding motifs (Fig. 3a). The consensus sequence for this tripartite binding motif is also present in the PP1-binding domain of RepoMan and is phylogenetically conserved (Fig. 3a,b). Mutation of either of these binding motifs abolished or decreased the binding of PP1 to ectopically expressed EGFP-RepoMan (Fig. 3c). The separate mutation of the two basic residues of the putative Arg motif showed that PP1-binding was only affected by mutation of K416. Thus, RepoMan has a functional tripartite PP1-binding domain.

The three PP1-binding motifs of RepoMan are each C-terminally flanked by a conserved SP or TP (Fig. 3b), representing putative phosphorylation motifs for Cdks. Consistent with previous data in DT40 cells^8^,^20^, RepoMan was hyperphosphorylated in mitotically arrested U2OS cells, as suggested by its reduced mobility during SDS–polyacrylamide gel electrophoresis (Fig. 3d). Mutation of the putative Cdk sites S400, T412 and T419 in the PP1-binding domain of RepoMan into an alanine, further referred to as RepoMan-3A, reduced this mobility shift and increased the binding of PP1. This suggested that Cdk1 opposes the RepoMan-PP1 interaction through phosphorylation of RepoMan at these sites. Phosphorylation of S400 and T412 have been detected in previous proteomics studies^14^,^24^,^25^. In addition, we raised a phospho-epitope-specific antibody against T412 of RepoMan. This residue was previously shown to be the major Cdk1 phosphorylation site that regulates the PP1-RepoMan interaction in DT40 cells^8^. The antibody visualized WT EGFP-RepoMan but not the T412A mutant in traps from mitotic cell lysates, confirming its specificity (Fig. 3e). In addition, the immunoblot signal was detected in lysates from mitotic cells but not from non-synchronized cells, supporting the notion that T412 was only phosphorylated during mitosis. In addition, T412 was phosphorylated *in vitro* by both Cdk2/Cyclin A (Supplementary Fig. 2) and Cdk1/Cyclin B. These Cdks have the same *in vitro* substrate specificity and are thus equivalent for biochemical studies^26^. Next, we generated phosphomimicking mutants for T412 separately (RepoMan-T412D) or for all three Cdk sites together (RepoMan-3D). RepoMan-T412D showed less PP1 binding but the 3D mutant caused a more prominent drop, indicating that the phosphorylation of S400 and/or T419 also contribute to the attenuation of PP1 binding (Fig. 3f,g). To further validate these findings, we phosphorylated RepoMan-wild type (WT) or RepoMan-3A from non-synchronized cells with Cdk2/Cyclin A (Fig. 3h). Cdk phosphorylation caused the dissociation of PP1 from RepoMan-WT but not from RepoMan-3A, confirming that phosphorylation of the Cdk sites in the PP1-binding region caused the disruption of the PP1-RepoMan complex (Fig. 3h).

The C-terminus of all PP1 isoforms contains a conserved Cdk phosphorylation site^7^. Phosphorylation of this site (PP1ph) reduces the catalytic efficiency of PP1. Using a phospho-epitope-specific antibody, RepoMan-associated PP1 was found to be phosphorylated in prometaphase-arrested U2OS cells (Fig. 3i). As expected, this phosphorylation was barely detected in RepoMan-associated PP1 from non-synchronized cells. Moreover, PP1 that was trapped with RepoMan-3A from lysates of non-synchronized cells could be readily phosphorylated *in vitro* (Fig. 3j). However, this does not imply that the Cdk1-mediated phosphorylation of PP1 is dependent on RepoMan. In conclusion, Cdk1 not only reduces the recruitment of PP1 but also phosphorylates RepoMan-associated PP1 to decrease its specific activity.

### Cdk1 promotes the binding of PP2A-B56 to RepoMan

**Figure 4:**
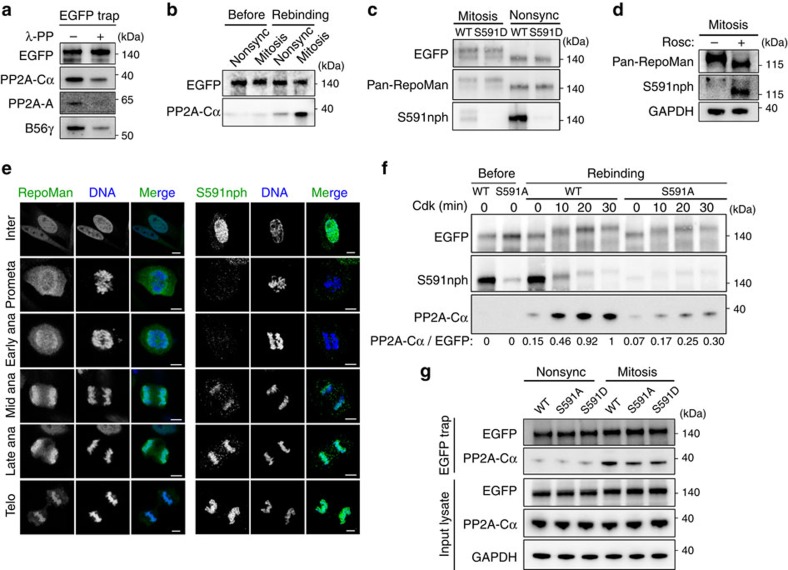
Cdk1 promotes the binding of PP2A-B56 to RepoMan. (**a**) Traps of EGFP-RepoMan-WT from Nocodazole-arrested U2OS cells were treated with or without λ-phosphatase for 30 min at 30 °C. The proteins released from the traps were washed away before the addition of SDS-sample buffer to the traps and immunoblot analysis for the presence of associated PP2A-B56 subunits. (**b**) Traps of EGFP-RepoMan-WT from non-synchronized or Nocodazole-arrested U2OS cells were washed with 1.5 M NaCl to remove associated PP2A (Before). Subsequently, the traps were added to freshly prepared mitotic lysates and asssociated PP2A was quantified by immunoblotting of the pelleted traps (Rebinding). (**c**) Cell lysates were prepared from Nocodazole-arrested (Mitosis) or non-synchronized (Nonsync) U2OS cells expressing EGFP-tagged RepoMan-WT or RepoMan-S591D. The EGFP traps were analysed by immunoblotting for EGFP, RepoMan (pan-RepoMan) or the phosphorylation of RepoMan at S591 (S591nph). (**d**) Nocodazole-arrested U2OS cells were treated with or without 100 μM Roscovitine for 1 h. The phosphorylation of S591 was examined by immunoblotting with the S591nph antibody. (**e**) Immunofluorescence staining of unperturbed U2OS cells at different stages of the cell cycle with the pan-RepoMan or S591nph antibodies. Scale bars represent 5 μm. (**f**) EGFP traps from lysates of non-synchronized HEK293T cells expressing EGFP-tagged RepoMan-WT or RepoMan-S591A were washed with 1.5 M NaCl to remove associated PP2A. Subsequently, the traps were phosphorylated with Cdk2 for the indicated time points and added to freshly prepared mitotic cell lysates from HeLa cells to examine PP2A binding to the pelleted traps. Phosphorylation of the fusions is suggested by mobility shifts and the loss of S591nph. (**g**) Traps of EGFP-tagged RepoMan-WT, RepoMan-S591A or RepoMan-S591D from non-synchronized (Nonsync) or Nocodazole-arrested (Mitosis) U2OS cells were analysed by immunoblotting for the binding of PP2A.

Next, we explored how Cdk1 regulates the interaction of RepoMan with PP2A-B56. Treatment of EGFP-RepoMan traps from mitotic U2OS cell lysates with lambda phosphatase decreased the binding of PP2A-B56 (Fig. 4a), indicating that the interaction between RepoMan and PP2A-B56 was phosphorylation-dependent. To identify which component was modified, we first trapped EGFP-tagged RepoMan from non-synchronized and mitotic cell lysates, and washed the traps with 1.5 M NaCl to remove associated PP2A. The purified fusions were then added back to mitotic lysates and examined for rebinding of PP2A. In these conditions, EGFP-RepoMan from mitotic lysates bound much more PP2A than that from lysates of non-synchronized cells (Fig. 4b). Since mitotic RepoMan was equally efficient in binding PP2A from interphase and mitotic lysates (Supplementary Fig. 3a), these data demonstrate that the RepoMan-PP2A interaction is enhanced by a mitosis-specific modification of RepoMan.

The kinetochore protein BubR1 contains a LSPI-type binding motif for the B56 subunit of PP2A that is phosphorylated by Cdk1 (refs 11, 13). S591 in the equivalent B56-binding motif of RepoMan also represents a consensus Cdk phosphorylation site^13^. To examine whether this site is phosphorylated during mitosis, we made an antibody against a synthetic peptide that includes S591. This antibody turned out to be specific for the non-phosphorylated (nph) peptide (Supplementary Fig. 3b). The S591nph-specific antibody visualized ectopically expressed EGFP-RepoMan on immunoblots from non-synchronized cell lysates but not from mitotic lysates (Fig. 4c). However, an interphase signal was absent after mutation of S591 (Fig. 4c) and mitotic RepoMan could be detected after Cdk1 inhibition (Fig. 4d). Immunostaining of fixed U2OS cells with the S591nph-specific antibody barely visualized RepoMan from prometaphase to early anaphase (Fig. 4e), consistent with its phosphorylation by Cdk1 *in vivo*. However, RepoMan was present throughout mitosis as it could be detected with a (phosphorylation-independent) pan-RepoMan antibody. Collectively, these data show that S591 is phosphorylated by Cdk1 and dephosphorylated again in early anaphase.

To delineate the effect of S591 phosphorylation on the recruitment of PP2A-B56, we purified EGFP-tagged RepoMan-WT and RepoMan-S591A from non-synchronized cells, phosphorylated the fusions *in vitro* with Cdk2 and examined the effect on PP2A binding from mitotic cell lysates. With progressing phosphorylation of EGFP-RepoMan-WT, as shown by mobility shifts and the disappearance of the S591nph signal, more PP2A was retained from mitotic lysates (Fig. 4f). The binding of PP2A was severely reduced with the S591A mutant, illustrating its dependency on S591 phosphorylation. However, this mutant still showed some Cdk-dependent phosphatase binding, suggesting that additional Cdk1 site(s) may contribute to the interaction with PP2A. Consistent with a Cdk-enhanced binding of PP2A, a synthetic LSPI-containing peptide coupled to Sepharose beads pulled down more PP2A from mitotic lysates when it was phosphorylated on S591 (Supplementary Fig. 3b). In contrast, Cdk phosphorylation of EGFP-PP2A-B56γ purified from non-synchronized cell lysates did not affect its binding to RepoMan from mitotic lysates (Supplementary Fig. 3c), indicating that Cdks do not promote the RepoMan/PP2A-B56 interaction through phosphorylation of the PP2A-B56 complex. In further agreement with these *in vitro* data, we found that the EGFP-RepoMan-S591A mutant showed a reduced binding of PP2A when trapped from mitotic cell lysates (Fig. 4g). However, the S591D mutant also bound less PP2A, indicating that this mutant is not phosphomimicking.

### Cdk1 inactivation makes RepoMan targeting irreversible

**Figure 5:**
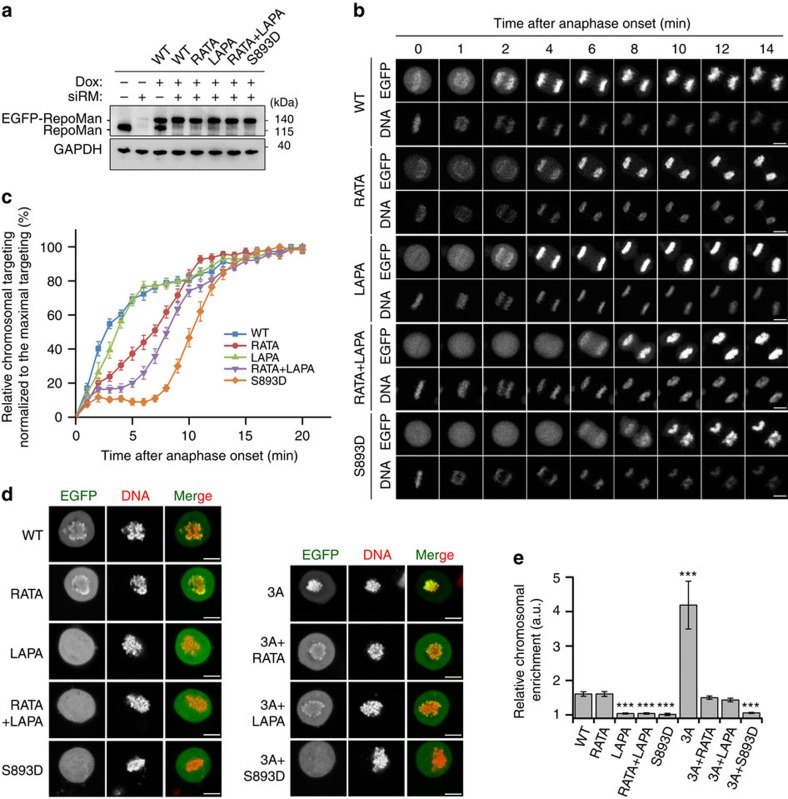
The chromosome targeting of RepoMan is regulated by both PP2A-B56 and PP1. (**a**) One day after the knockdown (siRM) of endogenous RepoMan in HeLa Flp-in T-Rex cells, siRNA-resistant EGFP-RepoMan fusions were induced for 24 h with doxycycline (Dox). Endogenous RepoMan and the EGFP-fusions were visualized by immunoblotting with pan-RepoMan antibodies. (**b**) Same as in **a** but the cells were subjected to time-lapse imaging by confocal microscopy after release from a 6 h-Monastrol arrest. The panel shows cells at different time points after the initiation of anaphase. DNA was stained with Hoechst 33342. Scale bars, 5 μm. (**c**) Quantification of the chromosome targeting (EGFP/DNA ratio) of the EGFP-RepoMan fusions as shown in (**b**). The means±s.e.m. of at least 11 cells are shown in each condition. Data were normalized to the maximal ratio of EGFP/DNA in each condition. (**d**) Live imaging of Nocodazole-arrested U2OS cells after expression of EGFP-tagged RepoMan or its phosphatase/histone-binding mutants. The figure shows the localization of DNA and the EGFP fusions. Scale bars, 5 μm. (**e**) Quantification of the ratio of EGFP signal on chromosome versus cytoplasm, as shown in **d**. The means±s.e.m. of at least 12 cells were analysed in each condition. ***P<0.001 in paired *t*-test, as compared with WT.

The bulk recruitment of RepoMan to chromosomes in early anaphase requires its dephosphorylation at S893 but coincides with the dissociation of PP2A-B56, indicating that another phosphatase dephosphorylates S893 at this stage. To identify the RepoMan-S893 phosphatase in anaphase, we generated stable HeLa cell lines (Flp-in/T-Rex system) that inducibly express siRNA-resistant EGFP-tagged fusions of RepoMan variants, that is, the WT protein, a PP1-binding mutant (RATA), a PP2A-binding mutant (LAPA), a PP1+PP2A-binding mutant (RATA+LAPA) and a histone-binding mutant (S893D). The RepoMan fusions were expressed to similar levels as endogenous RepoMan (Fig. 5a). Using time-lapse live imaging, we determined the kinetics of chromosome binding of the EGFP-RepoMan fusions during anaphase after the knockdown of endogenous RepoMan (Fig. 5a). The experiments were performed in cells that were released from a Monastrol-induced prometaphase arrest (Fig. 5b,c) and in unperturbed cells (Supplementary Fig. 4a), with a similar outcome.

Quantification of the data from Monastrol-released cells showed that the recruitment of RepoMan-WT started quickly after the onset of anaphase and was half-maximal after 2–3 min (Fig. 5c). The LAPA mutant was recruited almost as fast as the WT protein, while the RATA mutant showed a slower targeting, with half-maximal binding after 7–8 min. These data indicated that the chromosome targeting of RepoMan in early anaphase mainly depends on PP1, rather than PP2A-B56, coinciding with the increased recruitment of PP1 by RepoMan. This view was substantiated by *in vitro* data showing that a C-terminal RepoMan fragment that was phosphorylated at S893 could be dephosphorylated by purified PP1 and by EGFP-tagged RepoMan-WT and RepoMan-LAPA that were trapped from non-synchronized cells (Supplementary Fig. 4b). However, this dephosphorylation was not seen with the RepoMan-RATA mutant. Thus, the switch in the dephosphorylation of RepoMan-S893 from PP2A-B56 in (pro)metaphase to PP1 in anaphase makes the chromosome targeting of RepoMan Cdk1-independent and irreversible. Interestingly, the RATA+LAPA mutant showed a further delay in chromosome binding, as compared with the RATA mutant, suggesting that PP2A-B56 still promotes the chromosome targeting of RepoMan when the PP1-binding site is mutated. This is possibly due to a slower dissociation of PP2A-B56 from RepoMan RATA, as suggested by the delayed appearance of the S591nph signal in the RATA mutant (Supplementary Fig. 4c). However, further investigations are required to examine how PP1 contributes to the removal of PP2A-B56 from RepoMan in anaphase.

The histone-binding mutant (S893D) of RepoMan only started to co-localize massively with the chromosomes at ∼8 min after the beginning of anaphase (Fig. 5c). This corresponds to the time of initiation of nuclear import^27^ and therefore probably reflects RepoMan import rather than chromosome binding. Indeed, the S893D mutant only started to co-localize with the chromosomes when Lamin B1, a marker for nuclear import establishment, was already concentrated on the nuclear rim (Supplementary Fig. 4d). Moreover, C-terminal deletion mutants of RepoMan, which lack the histone-binding domain but still contain the Importin-β binding domain and putative consensus nuclear localization signals, were also targeted to the nucleus in telophase (Supplementary Fig. 4e).

In prometaphase cells the detection of chromosome association of RepoMan is lost by fixation but can be readily visualized by live imaging. RepoMan-RATA showed a normal, that is, partial chromosome localization in prometaphase, but RepoMan-LAPA was excluded from the chromosomes (Fig. 5d,e), in accordance with the dephosphorylation of RepoMan at S893 by associated PP2A-B56 (ref. 13). Since PP1 emerged as the major RepoMan-S893 phosphatase in anaphase, we speculated that an anaphase-like, chromosome hypertargeting of RepoMan could already be induced in prometaphase by the expression of RepoMan-3A, which bypasses the downregulation of PP1 binding by Cdk1. Indeed, RepoMan-3A was already massively chromosome-associated in prometaphase, as detected by live imaging (Fig. 5d,e). This hypertargeting phenotype was not seen with the double mutants RepoMan-3A+RATA and RepoMan-3A+S893D, indicating that it depended on both PP1 and the dephosphorylation of S893. Also, the hypertargeting was reduced with the RepoMan-3A+LAPA mutant, suggesting that it was mediated by both PP1 and PP2A-B56.

### Cdk1 activates Aurora B through inhibition of PP1-RepoMan

**Figure 6:**
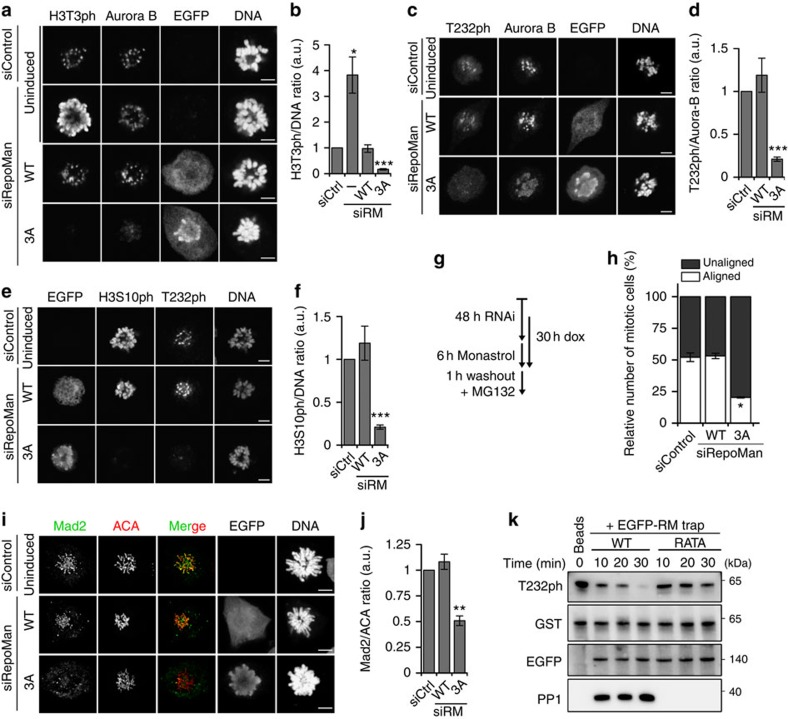
The inhibition of PP1-RepoMan maintains Aurora B activity in prometaphase. (**a**) HeLa Flp-in T-Rex cells were induced to express siRNA-resistant EGFP-tagged RepoMan-WT or RepoMan-3A following the knockdown of endogenous RepoMan. Monastrol-arrested mitotic cells were fixed before staining. Scale bars, 5 μm. (**b**) Quantification of the H3T3ph/DNA ratio in **a**. The data represent means±s.e.m. for three independent experiments (≥11 cells per condition in each experiment). (**c**) Cells were treated as in **a** but stained for Aurora B, Aurora-B -T232ph and DNA. Scale bars, 5 μm. (**d**) Quantification of the T232ph/Aurora-B ratio in **c**. The data represent means±s.e.m. for three independent experiments (≥11 cells per condition in each experiment) with paired *t*-test, as compared with siCtrl. (**e**) Cells were treated as in **a** but stained for H3S10ph, Aurora-B and DNA. Scale bars, 5 μm. (**f**) Quantification of the H3S10ph/DNA ratio in **e**. The data represent means±s.e.m. for three independent experiments (≥10 cells per condition in each experiment). (**g**) Cell-treatment scheme for chromosome alignment assays of HeLa Flp-in T-Rex cells, before and after induction of EGFP-tagged RepoMan-WT or RepoMan-3A, and following the knockdown of endogenous RepoMan. (**h**) In cells treated as explained in **g**, the percentage of cells with a well-aligned metaphase plate or unaligned chromosomes were plotted as mean percentages±s.e.m. for three independent experiments (≥121 cells per condition in each experiment). (**i**) Cells were treated as in **a** but stained for Mad2, ACA and DNA. (**j**) Quantification of relative signal of centromeric Mad2 in **i**. Data were normalized to the indicated ratio as means±s.e.m. for three independent experiments (≥8 centromeres per cell, ≥10 cells per condition in each experiment). (**k**) Traps of EGFP-fusions with RepoMan-WT or RepoMan-RATA from non-synchronized HEK293T cells were used as a phosphatase source to dephosphorylate recombinant GST-Aurora B/INCENP. The figure shows different time points of incubation with the traps analysed by immunoblotting. *P<0.05; **P<0.01; ***P<0.001, with paired *t*-test as compared with siCtrl.

As the downregulation of PP1-RepoMan by Cdk1 prevented the precocious bulk dephosphorylation of RepoMan at S893, we asked whether this also applies to other mitotic-exit substrates of PP1-RepoMan. In prometaphase-arrested Flp-in/T-Rex cells, the induction of RepoMan-WT rescued the H3T3 hyperphosphorylation phenotype caused by the knockdown of endogenous RepoMan (Fig. 6a,b and Supplementary Fig. 5a). However, the induction of RepoMan-3A also caused a premature dephosphorylation of centromeric H3T3 in prometaphase and, consequently, the loss of Aurora B and its substrate MCAK from the centromeres (Fig. 6a,b and Supplementary Fig. 5b,c). In addition, RepoMan-3A strongly reduced the Aurora-B activity, as indicated by its decreased autophosphorylation at T232 (Fig. 6c,d), and the decreased phosphorylation of histone H3 at S10 (H3S10ph), an established Aurora-B substrate on the chromosome arms (Fig. 6e,f). The loss of Aurora-B activity and H3S10ph cannot be solely explained by the reduced centromeric targeting of Aurora B via H3T3ph, since preventing H3T3 phosphorylation by the knockdown of Haspin affects neither the Aurora-B activity nor the level of H3S10ph (ref. 16). In further agreement with a loss of Aurora-B function, the expression of RepoMan-3A in the absence of endogenous RepoMan increased chromosome misalignment in cells that were released from a prometaphase arrest in the presence of MG132 (Fig. 6g,h). Aurora B is also implicated in the activation of the spindle checkpoint^28^. The expression of RepoMan-3A weakened the spindle checkpoint, as indicated by the reduced association of the checkpoint protein Mad2 with the kinetochores (Fig. 6i,j) and the override of the mitotic arrest in the presence of 3.3 μM nocodazole (Supplementary Fig. 5d). Since ectopically expressed RepoMan-3A bypasses Cdk1 regulation of associated PP1, these data demonstrate that restraining PP1-RepoMan is essential to maintain Aurora-B activity in (pro)metaphase.

Aurora B was also inactivated following the chromosome hypertargeting of endogenous RepoMan by Roscovitine. This Aurora-B inactivation was barely affected by the artificial tethering of Aurora B to the centromeres with CENPB-INCENP (Supplementary Fig. 5e,f), but was prevented by the addition of the phosphatase inhibitor Calyculin A (Supplementary Fig. 5g,h) suggesting that the inactivation of Aurora B was phosphatase-dependent. Since Cdk1 promotes Aurora B activation through multiple pathways (Supplementary Fig. 1), we have tried to delineate the contribution of PP1-RepoMan. The knockdown of RepoMan reduced the Roscovitine-induced Aurora B inactivation from ∼10 to 60% (Supplementary Fig. 5i,j). This effect was completely rescued by co-expression of siRNA-resistant WT EGFP-RepoMan, but only partially by the RepoMan-3D mutant. These data show that RepoMan is important for the control of Aurora B by Cdk1, but it cannot be excluded that this function of RepoMan is partially PP1-independent. Finally, we sought to examine whether PP1-RepoMan can directly dephosphorylate Aurora B. Indeed, the autophosphorylated Aurora B/INCENP complex was dephosphorylated by purified PP1 (Supplementary Fig. 5k) and RepoMan-WT isolated from lysates of non-synchronized cells (Fig. 6k). However, RepoMan-RATA failed to dephosphorylate this Aurora B complex, confirming that it required PP1.

### Cdk1 and PP1-RepoMan regulate the recruitment of Importin β

**Figure 7:**
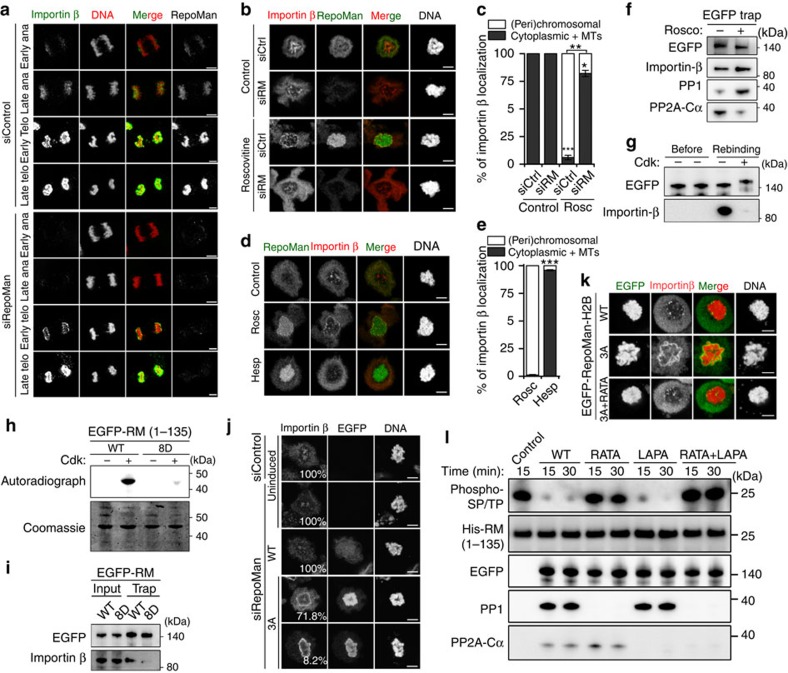
Cdk1 and PP1 antagonistically regulate the RepoMan/Importin β interaction. (**a**) U2OS cells transfected with control or RepoMan siRNA were fixed and stained after pre-extraction with 0.2% Triton X-100. Scale bars, 5 μm. (**b**) Nocodazole-arrested U2OS cells transfected with control or RepoMan siRNA were treated with DMSO or 100 μM Roscovitine for 30 min and subsequently stained. Scale bars, 5 μm. (**c**) Quantification for Importin-β localization as shown in **b**. The data are represented as mean percentages±s.e.m. for three independent experiments (≥66 cells per condition in each experiment). *P<0.05; **P<0.01; ***P<0.001 with paired *t*-test, as compared with siCtrl. (**d**) Nocodazole-arrested U2OS cells were treated with or without 100 μM Roscovitine (Rosc) or 200 nM Hesperadin (Hesp) for 30 min before staining. Scale bars, 5 μm. (**e**) Quantification for Importin-β localization as shown in **d**. The data are represented as mean percentages±s.e.m. for four independent experiments (≥76 cells per condition in each experiment). ***P<0.001 with paired *t*-test, as compared with Rosc. (**f**) Nocodazole-arrested U2OS cells expressing EGFP-RepoMan-WT were treated with DMSO or Roscovitine for 30 min. EGFP traps from the cell lysates were analysed by immunoblotting with the indicated antibodies. (**g**) EGFP traps from non-synchronized HEK293T cells expressing EGFP-RepoMan-WT were treated with 1.5 M NaCl, subjected to *in vitro* phosphorylation by Cdk2, and subsequently examined for Importin-β binding before or after incubation with mitotic cell lysates (Rebinding). (**h**) EGFP traps from HEK293T cells expressing EGFP-tagged RepoMan-(1–135)-WT or RepoMan-(1–135)-8D were radioactively phosphorylated with Cdk2 and analysed by autoradiography and Coomassie staining. (**i**) EGFP traps from HEK293T cells expressing EGFP-tagged RepoMan-WT or RepoMan-8D were analysed by immunoblotting for the binding of Importin β. (**j**) Nocodazole-arrested HeLa Flp-in T-Rex cells were transfected with control or RepoMan siRNAs, and induced to express EGFP-tagged RepoMan-WT or RepoMan-3A before staining. The numbers in the figures refer to the percentage of cells where the shown phenotype occurred. Scale bars, 5 μm. (**k**) Nocodazole-arrested Hela cells expressing H2B fusions of RepoMan-WT, RepoMan-3A and RepoMan-3A+RATA. Scale bars, 5 μm. (**l**) EGFP traps of the indicated RepoMan variants from non-synchronized HEK293T cells were used as a phosphatase source to dephosphorylate Cdk2-phosphorylated His-tagged RepoMan-(1–135).

The (peri)chromosomal recruitment of Importin β in anaphase, which functions as a seeding factor for the initiation of nuclear-envelope reassembly^29^, was reduced by the knockdown of RepoMan in DT40 cells^8^ and U2OS cells (Fig. 7a). This effect was lost in late telophase, suggesting that the recruitment of Importin β is biphasic and that RepoMan only affects the early recruitment of Importin β. Conversely, the Roscovitine-induced chromosome hypertargeting of RepoMan in prometaphase cells also caused a hypertargeting of Importin β to the chromosomes, which was alleviated by the knockdown of RepoMan (Fig. 7b,c). However, the inhibition of Aurora B with Hesperadin, which also endorses chromosome targeting of RepoMan in prometaphase, did not result in a premature recruitment of Importin β to the chromosomes (Fig. 7d,e). Similar data were obtained for the nucleoporin Nup153 (Supplementary Fig. 6a), which is an interactor of both Importin β and the N-terminus of RepoMan^8^. These data indicated that the chromosome recruitment of importin β in early anaphase depends on RepoMan and that the Importin-β/RepoMan interaction is opposed by Cdk1, consistent with previous data in DT40 cells^8^. Indeed, more Importin β was associated with EGFP-trapped RepoMan after the addition of Roscovitine to prometaphase-arrested cells (Fig. 7f). Also, purified interphase EGFP-tagged RepoMan retained much less Importin β from mitotic lysates after Cdk phosphorylation (Fig. 7g). In contrast, Cdk phosphorylation of EGFP-tagged Importin β did not affect its ability to bind RepoMan (Supplementary Fig. 6b).

The Importin-β binding domain of RepoMan (residues 1–135) contains eight potential Cdk phosphorylation sites (Supplementary Fig. 6c) and four of these are known to be hyperphosphorylated in mitosis^24^,^25^,^30^. When the eight putative Cdk1 sites were mutated into a phosphomimicking 8D mutant, RepoMan-(1–135) could no longer be phosphorylated by Cdk2 (Fig. 7h). Moreover, the corresponding full-length RepoMan-8D did not bind to Importin β in cell lysates (Fig. 7i). Alanine mutation of the N-terminal Cdk sites also reduced the binding of Importin β (data not shown), but further investigations revealed that this unexpected result can be explained by the requirement of one of these sites (Thr68) for Importin-β binding, as mutation of Thr68 to any of four tested residues compromised Importin-β binding (Supplementary Fig. 6d). Interestingly, changing the Cdk consensus by mutating Pro69 to Thr preserved the Importin-β binding (Supplementary Fig. 6d).

Since inactivation of Cdk1 promotes the RepoMan/Importin β interaction, which is correlated with an increased PP1-RepoMan interaction, we hypothesized that the Importin-β binding site of RepoMan is a substrate for associated PP1. Accordingly, replacement of endogenous RepoMan by RepoMan-3A, which is hypertargeted to chromosomes and is constitutively associated with PP1, induced a partial or complete premature (peri)chromosome recruitment of Importin β in Nocodazole-arrested cells (Fig. 7j). However, the forced targeting of RepoMan-WT to prometaphase chromosomes by its fusion with histone H2B did not cause the co-recruitment of Importin β in prometaphase (Fig. 7k). This showed that chromosome targeting of RepoMan *per se*, even when combined with prometaphase levels of associated PP1, was not sufficient for the co-recruitment of Importin β. However, the prometaphase recruitment of Importin β induced by the expression of H2B fused to RepoMan-3A was lost by mutation of the RVxF-type PP1 binding motif, revealing its PP1 dependency. Interestingly, we found that RepoMan-3A also induced a premature association of Nup153, another early nuclear-envelope seeding factor, to the (peri)chromosomal area during anaphase (Supplementary Fig. 6e). In contrast, RepoMan-3A did not affect the distribution of Lamin B1, which marks the end of nuclear-envelope reassembly (Supplementary Fig. 6f). These data indicate that the RepoMan pathway is only one of multiple processes that are involved in nuclear-envelope reassembly. Finally, purified PP1 (Supplementary Fig. 6g) and EGFP-tagged PP1-RepoMan (Fig. 7l) trapped from non-synchronized cells were able to dephosphorylate Cdk-phosphorylated RepoMan-(1–135) *in vitro*. The dephosphorylation was specific for PP1 as it was lost by mutation of the PP1-binding site but not by mutation of the PP2A-binding site. Collectively, these data strongly suggest that Cdk1 and PP1 antagonistically regulate the binding of Importin β to RepoMan. However, it should be noted that the localization of RepoMan and Importin β were not completely identical after the inhibition of Cdk1 or the expression of RepoMan-3A (Fig. 7b,d,j,k), in that RepoMan was more prominently associated with the chromosomes, whereas Importin β was preferentially targeted to the chromosomal periphery. It therefore seems likely that the RepoMan-Importin β interaction is somehow opposed on the chromosome and/or that PP1-RepoMan dephosphorylates additional substrates to enhance the association of Importin β with the chromosomal periphery.

## Discussion

**Figure 8:**
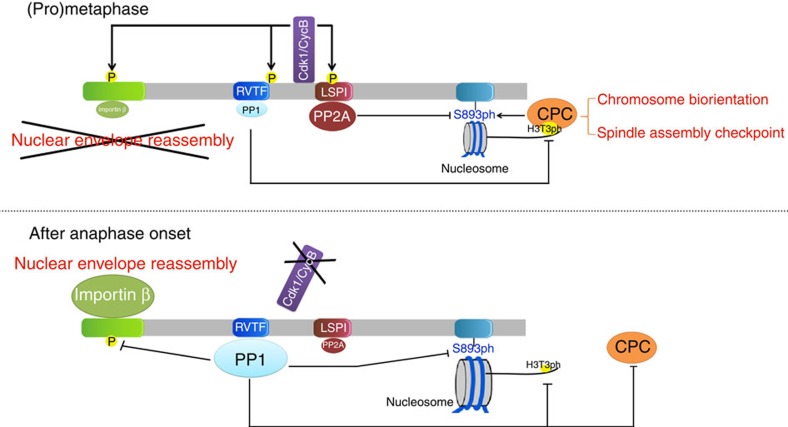
Model of the Cdk1-mediated coordination of RepoMan-associated phosphatases. During (pro)metaphase, Cdk1 promotes Aurora-B activation and prevents nuclear-envelope reassembly through phosphorylation of RepoMan at multiple sites, which reduces the binding of PP1 and Importin β. In contrast, the Cdk1-mediated phosphorylation of RepoMan at S591 enhances the recruitment of PP2A, which contributes to the dynamic chromosome targeting of RepoMan in (pro)metaphase and the centromeric localization of Aurora B. After anaphase onset, Cdk1 inactivation leads to the dephosphorylation of RepoMan, associated with the loss of PP2A and the bulk recruitment of PP1-RepoMan to the chromosomes. PP1-RepoMan then inactivates Aurora B and promotes nuclear-envelope reassembly through dephosphorylation of Histone H3 at T3, Aurora B at T232 and RepoMan at S893 and the Importin-β-binding domain.

Our data revealed that the regulation of RepoMan-associated phosphatases by Cdk1 is critical for the ordered dephosphorylation of their substrates in (pro)metaphase and anaphase (Fig. 8). On the one hand, Cdk1 promotes the recruitment of PP2A-B56 to reverse the Aurora-B-mediated phosphorylation of RepoMan at S893 in the histone-binding site (Fig. 4). Hence, it is the balance between Aurora B and PP2A-B56 that determines how much RepoMan is chromosome-associated during (pro)metaphase. This is probably a highly dynamic process because Cdk1 also stimulates the recruitment of Aurora B to the centromeres (Supplementary Fig. 1), which limits the chromosome targeting of RepoMan. On the other hand, Cdk1 reduces the binding of PP1 to RepoMan and phosphorylates associated PP1 (Fig. 3), which is known to decrease its catalytic efficiency^7^. The remaining pool of chromosome-associated PP1-RepoMan is essential and sufficient to dephosphorylate H3T3 on the chromosome arms, in agreement with the finding that H3T3ph is an extremely good substrate for PP1-RepoMan, even when compared with other histone H3 phosphorylation sites^15^. The preferential dephosphorylation of histone H3 at Thr3 is also in accordance with compelling data showing that the substrate quality governs the order of protein dephosphorylation by mitotic phosphatases^31^. Centromeric H3T3ph is protected from dephosphorylation during (pro)metaphase because the recruitment of PP1-RepoMan is locally opposed by Aurora B, which is most active at the centromeres^13^. The significance of the Cdk1-mediated downregulation of chromosome-associated PP1-RepoMan is probably that it prevents the dephosphorylation of substrates, including Aurora B and the histone/importin-β- binding sites of RepoMan, that only need to be dephosphorylated in anaphase. Consistent with this proposal, we found that bypassing the Cdk1 regulation of PP1-RepoMan, either by the expression of RepoMan-3A or inhibition of Cdk1, caused the premature dephosphorylation of these substrates in prometaphase (Figs 5, 6, 7).

The inactivation of Cdk1 in early anaphase induces a phosphatase switch in that PP2A-B56 is dissociated from RepoMan, whereas PP1 is recruited more prominently (Figs 2 and 8). Moreover, RepoMan-associated PP1 is expected to become more active through autocatalytic dephosphorylation of its Cdk-phosphorylated C-terminus^7^. This phosphatase switch in early anaphase ends the collaboration between PP2A-B56 and PP1 that contributed to the centromeric targeting of the CPC through dephosphorylation of RepoMan-S893 and H3T3, respectively. However, this does not limit mitotic progression because the CPC translocates to the spindle midzone in anaphase and the dephosphorylation of RepoMan-S893 is taken over by PP1. Thus, S893 is a common substrate of RepoMan-associated PP2A-B56 and PP1, but at distinct phases of mitosis. The consecutive dephosphorylation of a protein by distinct phosphatases may be a more common feature of mitosis. Possibly, it also accounts for discrepant findings on the relative contribution of PP1 and PP2A-B56 to the dephosphorylation of the kinetochore protein KNL1 (refs 32, 33).

The PP1-mediated dephosphorylation of RepoMan at S893 in anaphase is not restrained by Cdk1, which is no longer active and is less efficiently counteracted by Aurora B after its translocation to the spindle midzone (Fig. 8). Hence, the phosphatase switch in anaphase makes the chromosome targeting of RepoMan largely kinase-independent and irreversible, culminating in its bulk recruitment to the chromosomes in anaphase. However, it should be noted that even after its translocation to the spindle midzone Aurora B still affects chromosome-associated processes through a midzone gradient that is opposed by RepoMan, probably through associated PP1 (refs 21, 34). PP1-RepoMan also becomes sufficiently active in anaphase to cause a net dephosphorylation of the Importin-β-binding sites of RepoMan and Aurora B at the chromosomes. It is possible that PP1-RepoMan also dephosphorylates chromosome-associated substrates of Aurora B, as suggested previously^8^,^21^, but our data do not allow us to differentiate between effects on Aurora B and/or its substrates.

Recently, PP1-PP2A activation relay systems have been described for the mitotic exit in fission yeast^35^ and spindle-assembly checkpoint silencing in human cells^33^. The PP2A-PP1 switch on RepoMan in our study presents a new type of phosphatase relay. However, at present it is not clear which phosphatase dephosphorylates the Cdk1 sites in the PP1 and PP2A-binding domains of RepoMan and induces the anaphase switch in phosphatase binding. Our preliminary data suggest that the activation of this ‘priming' phosphatase not only requires the inactivation of Cdk1 but also depends on a proteolytic event (unpublished data), consistent with the existence of a hitherto unidentified proteasome-activated mitotic-exit phosphatase^36^.

In conclusion, we have found that the activities of RepoMan-associated PP2A-B56 and PP1 are coordinated by Cdk1 and that this is essential for the ordered dephosphorylation of their substrates in mitosis.

## Methods

### Materials

Antibodies against EGFP (SC-8334, Santa Cruz, 1:1,000), GAPDH (2118, Cell Signaling, 1:5,000), H3S10ph (9706, Cell Signaling, 1:1,000), ACA (HCT-0100, ImmunoVision, 1:4,000), Aurora B (611082, BD Transduction Laboratories, 1:250), H3T3ph (07–424, Millipore, 1:10,000), RepoMan (HPA030049, Sigma, 1:300 for IF, 1:1,000 for WB; Ab45129, Abcam, 1:1,000 for WB), PP2A-Cα (610556, BD Transduction Laboratories, 1:2,000), PP2A-B56γ (sc-67038, Santa Cruz, 1:1,000), Lamin B1 (sc-6216, Santa Cruz, 1:500), phospho-PP1 (2581S, Cell Signaling, 1:1,000), Importin β (ab2811, Abcam, 1:10,000 for IF, 1:2,000 for WB) and Nup153 (ab84872, abcam, 1:5,00) and phospho-Ser/Thr-Pro, also known as mpm2 (Merckmillipore, 05–368, 1:1,000) were purchased. MCAK antibodies were a gift from Dr Linda Wordeman (University of Washington School of Medicine). Swine anti-rabbit IgG-HRP (P0217, Dako,1:5,000), rabbit anti-mouse IgG-HRP (P0260, Dako,1:5,000), rabbit anti-goat IgG-HRP (P0160, Dako,1:5,000), donkey anti-mouse, goat anti-rabbit IgG-Alexa Fluor 488 (A11008, ab150109 from Invitrogen,1:1,000), goat anti-mouse, goat anti-rabbit IgG-Alexa Fluor 546 (A11030, A11010 from Invitrogen,1:1,000) and goat anti-mouse, goat anti-rabbit, goat anti-human IgG-Alexa Fluor 633 (A21052, A21070 and A21091 from Invitrogen,1:1,000) were also from commercial sources. A non-isoform-specific PP1 antibody and a RepoMan phospho-S893 antibody were made in house^13^,^37^ For the RepoMan S593nph antibody, a synthetic peptide comprising residues 581–599 of RepoMan was coupled to keyhole limpet haemocyanin or bovine serum albumin (BSA) (Imject Maleimide-activated keyhole limpet hemocyanin/BSA kit, Pierce) and used to generate antibody in rabbits. Similarly, the synthetic peptide LPANT(ph)PLRKGGC was used to generate a RepoMan T412ph antibody in rabbits. Calyculin A (EI-192, Biomol), Roscovitine (R-1234, LC Laboratories), Hesperadin (S1529, Selleckchem), Monastrol (orb146117, Biorbyt) and Microcystin-LR (10007188, Cayman) were purchased. Duplexes of siRNA against RepoMan (TGACAGACTTGACCAGAAA) and control siRNA (TAAGGCTATGAAGAGATAC) were obtained from Dharmacon. An siRNA-resistant EGFP-RepoMan construct was generated with primers 5′-GAG TCC GAG ATG ACG GAC TTA ACT AGG AAG GAA GGT CTC AGC-3′ and 5′-GCT GAG ACC TTC CTT CCT AGT TAA GTC CGT CAT CTC GGA CTC-3′. The indicated mutants were generated according to the QuickChange Mutagenesis Kit (Agilent Technologies) and verified by sequencing. Importin β1-EGFP and EGFP-CENPB-INCENP constructs were kind gifts from Dr Patrizia Lavia (Institute of Molecular Biology and Pathology, Italy) and Dr Susanne Lens (University Medical Center Utrecht, Netherland), respectively.

### Cell culture and transfections

HEK293T cells were cultured in high-glucose DMEM, supplemented with 10% fetal calf serum (FCS). U2OS human osteosarcoma cells were cultured in McCoy's 5A medium with 10% FCS. HeLa cells were cultured in low-glucose DMEM, supplemented with 10% FCS. All media contained penicillin and streptomycin. Transfection with plasmid DNA was carried out with X-tremeGENE 9 (Roche Applied Science) or jetPRIME (Polyplus Transfection) transfection Reagent. The siRNA transfections or co-transfection of plasmid DNA and siRNA were performed using jetPRIME transfection reagent for 48–60 h. Flp-In T-REx HeLa cells used for generating stable doxycycline-inducible cell lines were a gift from Dr Jonathon Pines (University of Cambridge, England, UK). Flp-In T-REx HeLa host cell lines were maintained in DMEM with 10% tetracycline-free fetal bovine serum supplemented with 50 μg ml^−1^ Zeocin. siRNA-resistant constructs encoding EGFP-RepoMan variants were cloned into pCDNA5/FRT/TO vectors (Invitrogen). All HeLa Flp-in cells stably expressing a doxycycline-inducible construct of EGFP-RepoMan were derived from the HeLa Flp-in host cell line by transfection with the pCDNA5/FRT/TO vector and pOG44 (Invitrogen), and cultured in the same medium but containing 200 μg ml^−1^ hygromycin and 4 μg ml^−1^ blasticidin. Gene expression was induced with 10 ng ml^−1^ doxycycline (Sigma-Aldrich) for 24 h or as indicated. Unless indicated otherwise, a prometaphase arrest was induced by culturing U2OS, HeLa, Hela Flp-in TRex or HEK293T cells consecutively for 24 h with 2 mM thymidine, 2 h without thymidine and 16 h with 100 ng ml^−1^ nocodazole or 100 μM Monastrol. The arrested U2OS and HeLa cells were harvested by shake-off.

### Biochemical procedures

For EGFP traps the soluble fraction (S1) after cell lysis and the chromatin-enriched fraction (S2) after micrococcal nuclease (300 U ml^−1^) treatment were collected^37^. The rest pellet after the micrococcal nuclease treatment was sonicated on ice water for 10 min, and the supernatant (S3) was combined with S1 and S2 for EGFP trapping of RepoMan fusions^37^. Similar cell lysates was prepared for immunoprecipitations of endogenous RepoMan (HPA030049,1:100). Of note, to preserve the phosphatase activity, the micrococcal nuclease treatment step was omitted in the EGFP-RepoMan trap when the S893D mutation was introduced (Figs 6k and 7l), and the sonication was shortened to 5 min. SDS–polyacrylamide gel electrophoresis was performed with 4–12%, 10% Bis-Tris or 3–8% Tris-acetate gels (NuPAGE, Invitrogen) for Coomassie brilliant blue staining or immunoblotting. For peptide pulldown, synthetic RepoMan-(581–599), either unmodified or phosphorylated at S591, was coupled to Maleimide-activated BSA (Pierce) via an additional C-terminal Cys residue. BSA or the BSA-linked peptide was coupled to CNBr-activated Sepharose-4B (GE Healthcare). The matrix was incubated with U2OS cell lysates. After washing with Tris-buffered solution supplemented with 0.2 M LiCl, the retained proteins were analysed for the presence of PP2A by immunoblotting. Recombinant GST-tagged Aurora-B-(1–344) fused to INCENP-(835–903) was expressed in bacteria and affinity purified^13^. Poly-His tagged fragments were expressed in bacteria and purified on Ni^2+^-Sepharose. For *in vitro* phosphorylation, purified RepoMan-(865–925) was incubated for 60 min at 30 °C with recombinant GST-AuroraB-INCENP in a buffer containing 20 mM Tris/HCl at pH 7.5, 2 mM dithiothreitol (DTT), 0.1 mg ml^−1^ BSA, 2 mM MgAc and 1 mM ATP. For *in vitro* phosphorylation with Cdks, samples were incubated for different time points at 30 °C with recombinant Cdk1/Cyclin B1 (0134–0135–1, Proqinase) or Cdk2/Cyclin A (homemade) in a buffer containing 20 mM Tris/HCl at pH 7.5, 2 mM DTT, 0.1 mg ml^−1^ BSA, 2 mM MgAc and 1 mM ATP or 100 μM γ^32^P-labelled ATP. Microcystin (0.5 μM) was added if no further dephosphorylation assay was followed. For *in vitro* dephosphorylation, 1 μg of Aurora-B-phosphorylated His-RepoMan-(865–925) or Cdk-phosphorylated His-RepoMan-(1–135) was incubated for indicated time points at 30 °C in a shaker with PP1 purified from rabbit skeletal muscle or an EGFP trap of the indicated fusions in a buffer containing 20 mM Tris/HCl at pH 7.5, 0.1 mg ml^−1^ BSA and 1 mM DTT in a total volume of 40 μl. Immunoblots were visualized using ECL reagent (Perkin Elmer) in an ImageQuant LAS4000 imaging system (GE Healthcare). Uncropped versions of blots appear in Supplementary Fig. 7. Coomassie brilliant blue stained radioactive gels were visualized with the Typhoon FLA 9500 system (GE Healthcare).

### Immunofluorescence staining and microscopy

For immunofluorescence studies, cells were consecutively grown on polylysine-coated coverslips in a 24-well chamber, fixed with 4% paraformaldehyde, permeabilized with 0.5% Triton X-100, blocked in 3% BSA/PBS and incubated in 1.5% BSA/PBS with the primary antibodies overnight at 4 °C, and with secondary antibodies for 1 h at room temperature. The DNA was stained with DAPI (fixed) or Hoechst 33342 (live imaging). Confocal images were acquired with a Leica TCS SPE laser-scanning confocal system mounted on a Leica DMI 4000B microscope and equipped with a Leica ACS APO 63X 1.30NA oil DIC objective. For live cell imaging, the Leica TCS SPE laser-scanning confocal microscope was further equipped with a live-imaging chamber ensuring 37 °C and 5% CO_2_, and a monochrome digital camera DFC365 FX from Leica. For time-lapse imaging, cells were grown in a four-well chambered coverglass (LabTek, Thermo Fisher Scientific). All immunofluorescence images of similarly stained experiments were acquired with identical illumination settings. The brightness and contrast were adjusted using only linear operations applied to the entire image. Final images were processed and assembled using Photoshop CS3 (Adobe). For quantification, Z-stack scans were performed through each cell (8–10 sections with 1 μm intervals for H3T3ph, H3S10ph and Aurora B T232ph; five sections with 0.5 μm intervals for MCAK and Mad2), and analysed using ImageJ software with the ‘sum slices' feature of Z project. MCAK and Mad2 fluorescence intensity was quantified for individual kinetochores selected manually by ACA staining after subtraction of the background signal (estimated from regions of the cell without kinetochores). The normalized ratio against ACA was plotted with Origin 8.5 software (OriginLab software).

## Additional information

**How to cite this article:** Qian, J. *et al.* Cdk1 orders mitotic events through coordination of a chromosome-associated phosphatase switch. *Nat. Commun.* 6:10215 doi: 10.1038/ncomms10215 (2015).

## Supplementary Material

Supplementary Information Supplementary Figures 1-7
