# Supplementary material for: Cdk1 orders mitotic events through coordination of a chromosome-associated phosphatase switch
Source: Nat Commun. 2015 Dec 17;6:10215. doi: 10.1038/ncomms10215 (PMC4703885; doi:10.1038/ncomms10215)
Supplement: Supplementary Information — Supplementary Figures 1-7 [file ncomms10215-s1.pdf]

Supplementary Figure 1

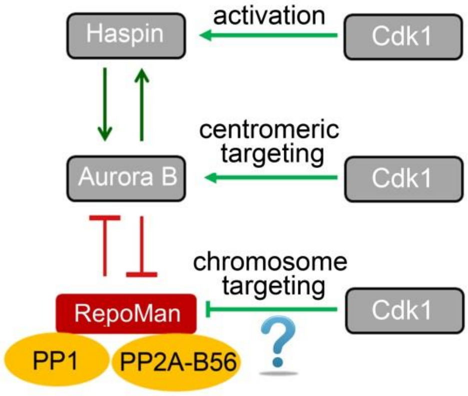


**Supplementary Figure 1. Scheme of the regulation of the centromeric targeting of Aurora B during (pro)metaphase.**

See the Introduction for relevant references.

Supplementary Figure 2

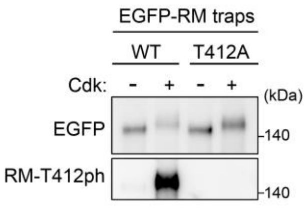


**Supplementary Figure 2. Cdk phosphorylates RepoMan at T412 *in vitro*.**

EGFP traps of RepoMan-WT or RepoMan-T412A from non-synchronized HEK293T cells were incubated with (+) or without (-) Cdk2/Cyclin A in the presence of MgATP before immunoblotting with phospho-T412 antibodies.

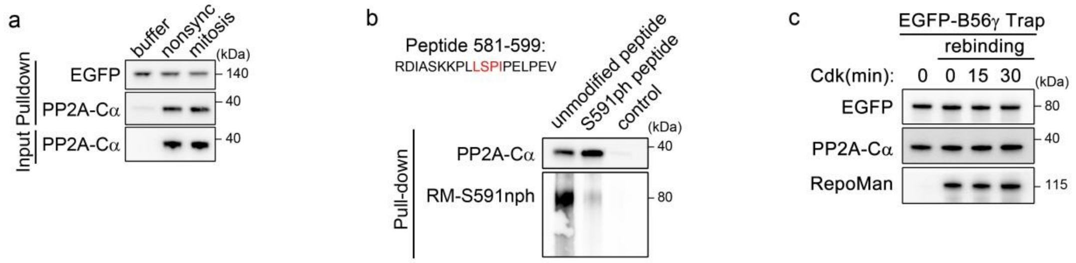


**Supplementary Figure 3. Regulation of the RepoMan/PP2A-B56 interaction**

(**a**) EGFP-traps of RepoMan from Nocodazole-arrested U2OS cells were washed with 1.5 M NaCl to remove PP2A, added to lysates from non-synchronized (nonsync) of prometaphase-arrested (mitosis) U2OS cells, and examined for binding of PP2A by immunoblotting of the pelleted traps. (**b**) A synthetic peptide comprising RepoMan-residues 581–599, either unmodified or phosphorylated at S591, was coupled to BSA. The peptide-BSA complex (peptide) or BSA alone (control) were subsequently linked to CNBr-activated Sepharose-4B and incubated for 30 min with U2OS cell lysates. The retained PP2A subunits on the pelleted beads were visualized by immunoblotting. (**c**) Non-synchronized HEK293T cells that transiently expressed EGFP-B56γ were used as a source for the purification of PP2A-B56γ by EGFP trapping from a lysate. The phosphatase was phosphorylated by Cdk2 *in vitro* for the indicated time points and examined for its ability to bind RepoMan from mitotic U2OS cell lysates in pulldown experiments (rebinding).

Supplementary Figure 4

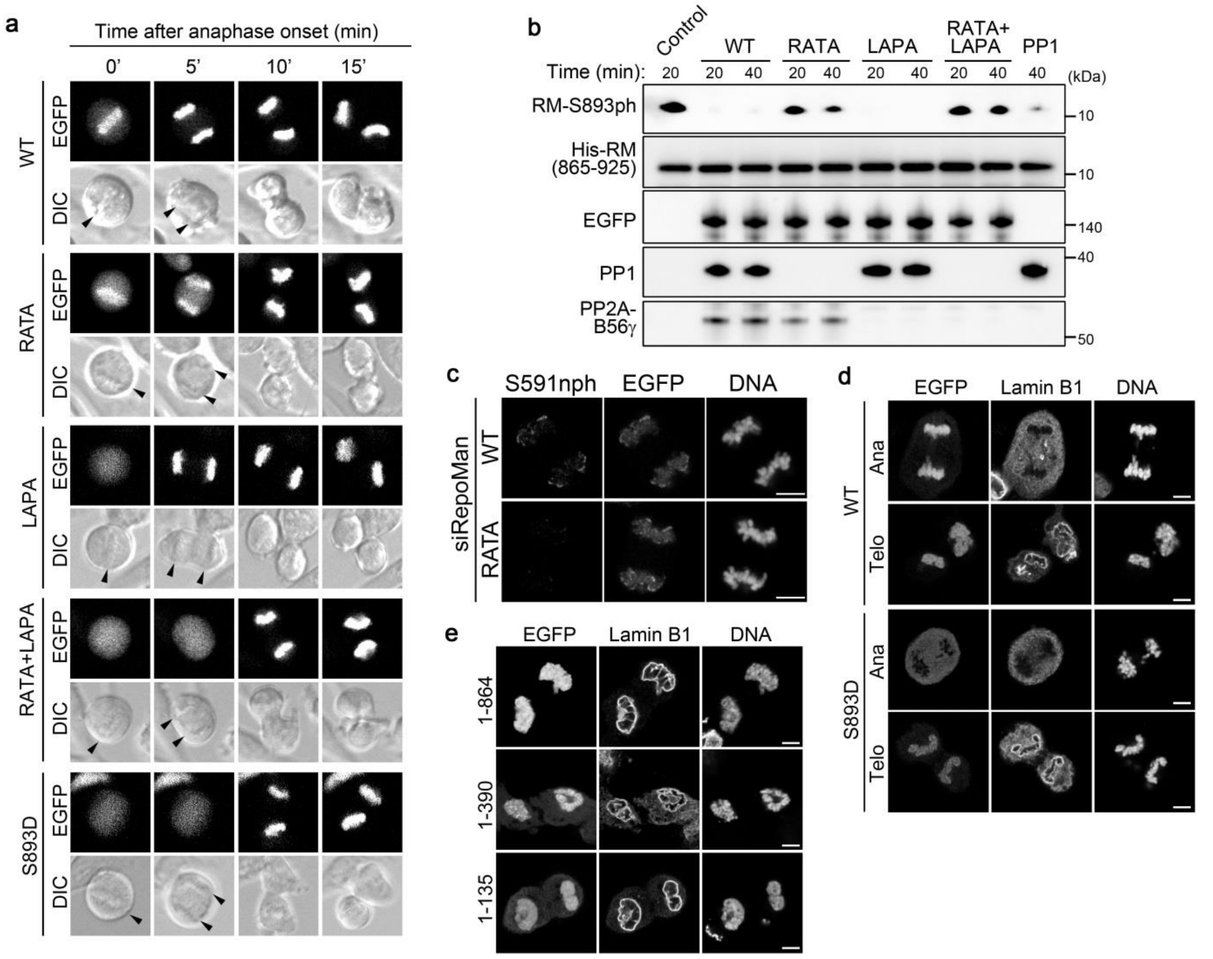


**Supplementary Figure 4. The chromosome targeting of RepoMan requires both PP2A-B56 and PP1**

(**a**) Hela Flp-in T-Rex cells were induced with doxycycline (Dox) for 24h to express siRNA-resistant EGFP-RepoMan-WT or the indicated fusion mutants. Endogenous RepoMan was knocked down 24h before the addition of Dox. The non-synchronized cells were subjected to time-lapse imaging. The figure shows DIC and EGFP signals at the indicated times after the onset of anaphase. (**b**) PP1 (180 nM) purified from rabbit skeletal muscle or EGFP traps of the indicated RepoMan variants from non-synchronized HEK293T cells were used to dephosphorylate His-tagged RepoMan-(865-925) that had been phosphorylated by Aurora B *in vitro*. Empty EGFP-trap beads were used as control. A S893D mutation was introduced into each variant to

avoid the nuclease-treatment step, which was found to reduce the activity of RepoMan-associated phosphatases. After incubation for the indicated times, EGFP-RepoMan, PP1, PP2A-B56γ and the phosphorylation level of RepoMan-S893 (RM-S893ph) were detected by immunoblotting. (**c**) HeLa Flp-in T-Rex cells were treated with siRNA for endogenous RepoMan and induced to express EGFP-RepoMan WT or RATA mutant. The immunostainings show early anaphase cells. (**d**) Similar to (**c**) but after induction of EGFP-RepoMan WT or S893D in anaphase or telophase. (**e**) U2OS cells in telophase that transiently expressed the indicated RepoMan fragments were subjected to immunostaining before imaging. Scale bars represent 5 μm.

Supplementary Figure 5

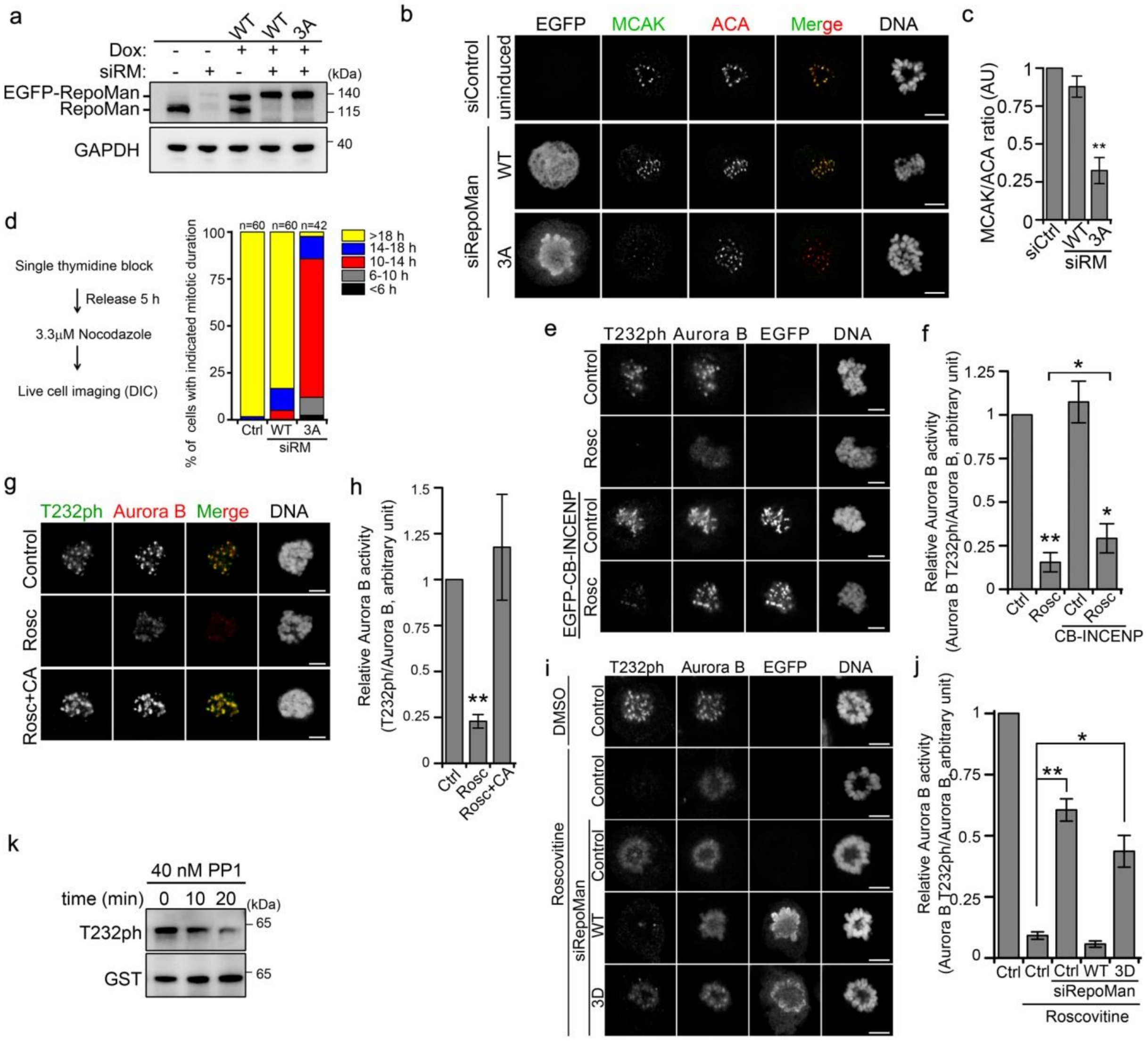


**Supplementary Figure 5. RepoMan-3A causes a dephosphorylation of Aurora B (substrates)**

(**a**) One day after the knockdown (siRM) of endogenous RepoMan in HeLa Flp-in T-Rex cells, siRNA-resistant EGFP-tagged RepoMan-WT or RepoMan-3A were induced for 24 h with doxycycline (Dox). Endogenous RepoMan and the EGFP-fusions were visualized by immunoblotting with pan-RepoMan antibodies. GAPDH was used as a loading control. (**b**) HeLa Flp-in T-Rex cells were either uninduced or induced to express siRNA-resistant EGFP-tagged RepoMan-WT or RepoMan-3A, after transfection with a control siRNA (siControl) or a siRNA for the knockdown of

endogenous RepoMan (siRepoMan). Monastrol-arrested mitotic cells were fixed before visualization of EGFP, MCAK, ACA and DNA. Scale bars represent 5 μm in all panels of Supplementary Fig. 5. (**c**) Quantification of the MCAK/ACA ratio in (**b**). The data represent means ± SEM for three independent experiments (≥ 11 cells per condition in each experiment). **, $P < 0.01$ with paired t-test, as compared to control siRNA (siCtrl). (**d**) Similar to (**b**) but synchronized according to the scheme indicated in the left panel and imaged with live microscopy for 28 hr in the presence of 3.3μM nocodazole. The right panel shows the time the cells spent in mitosis. (**e**) Nocodazole-arrested U2OS cells, transiently expressing EGFP-RepoMan-WT, with or without the co-expression of EGFP-tagged CENP-B (CB) fused to INCENP, were treated with DMSO (control) or Roscovitine (100 μM) for 30 min before fixation, immunofluorescence staining and confocal imaging. (**f**) Quantification of the T232ph/Aurora B ratio in (**e**). The data represent means ± SEM for three independent experiments (≥ 11 cells per condition in each experiment). *, $P < 0.05$; **, $P < 0.01$ with paired t-test, as compared to control (Ctrl) or between Roscovitine conditions. (**g**) Nocodazole-arrested U2OS cells were treated with DMSO (control) or 100 μM Roscovitine for 30 min, as such or combined with 20 nM Calyculin A. The fixed cells were stained for T232ph, Aurora B and DNA. (**h**) Quantification of the T232ph/Aurora B ratio in (**g**). The data represent means ± SEM for three independent experiments (≥ 15 cells per condition in each experiment). **, $P < 0.01$ with paired t-test, as compared to control (Ctrl). (**i**) Mitotically-arrested U2OS cells expressing EGFP-RepoMan WT or 3D, treated or not with siRNA for endogenous RepoMan, were treated with 100 μM Roscovitine for 30 min before fixation and immunostaining. (**j**) Quantification of the T232ph/Aurora B ratio in (**i**). The data represent means ± SEM for three independent experiments (⩾ 11 cells per condition in each experiment). **,

P < 0.01; *, P<0.05, with paired t-test (**k**) Recombinant, autophosphorylated GST-Aurora B/INCENP was incubated at 30 °C with 40 nM PP1 purified from rabbit skeletal muscle. The dephosphorylion was followed with T232ph antibodies.

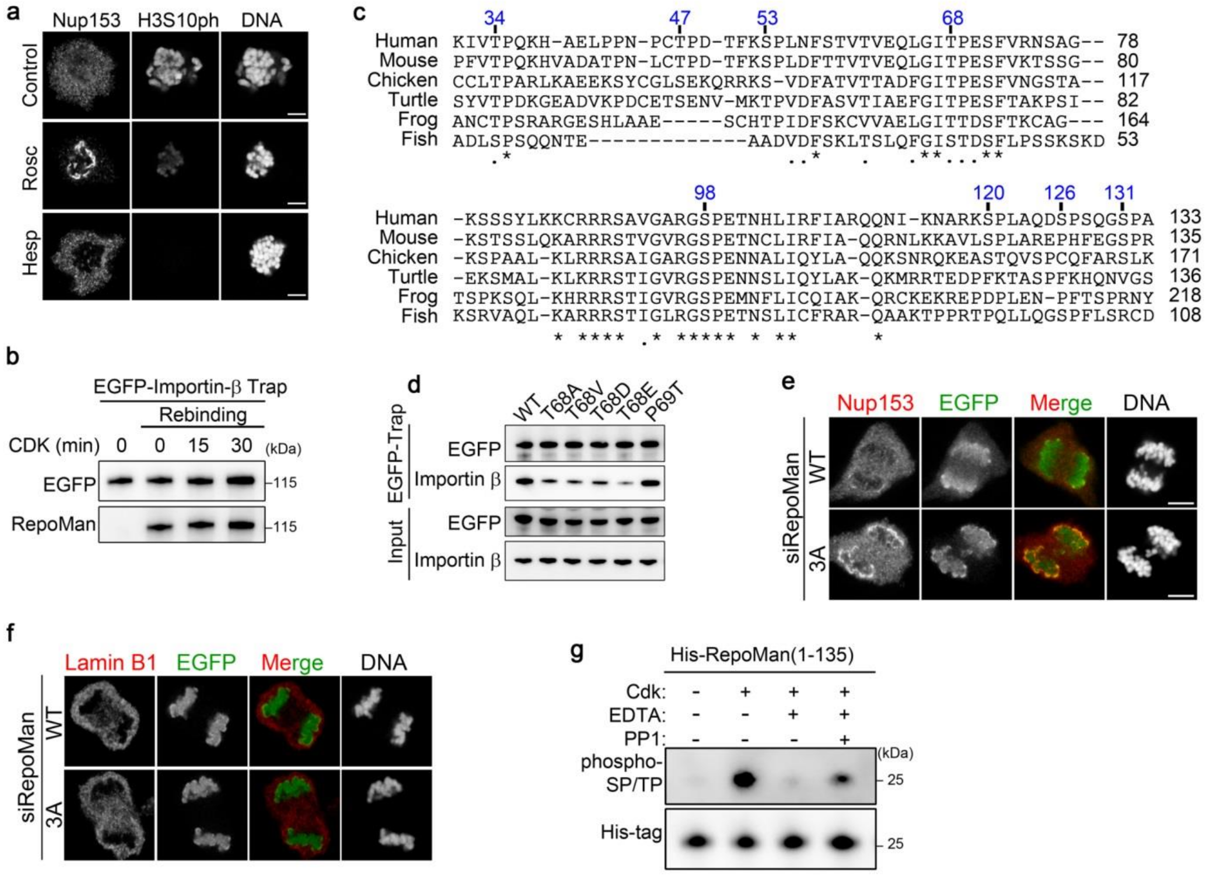

**Supplementary Figure 6. Cdk1 regulates the Importin β / RepoMan interaction**

(**a**) Nocodazole-arrested U2OS cells were treated with or without 100 μM Roscovitine or 200 nM Hesperadin for 30 min. The fixed cells were stained for Nup153 and H3S10ph. Scale bars represent 5 μm. (**b**) EGFP traps from non-synchronized HEK293T cells expressing EGFP-tagged Importin β were washed with 1.5 M NaCl. Subsequently, the washed traps were phosphorylated *in vitro* with Cdk2/cyclin A and added to mitotic HeLa cell lysates to examine the amount of RepoMan that co-sedimented with the traps (rebinding). (**c**) Sequence alignment of the N-terminus of RepoMan in vertebrates. The Cdk consensus phosphorylation sites are numbered in blue. (**d**) EGFP traps from non-synchronized HEK293T cells expressing EGFP-tagged RepoMan-WT or the indicated mutants were analyzed for the binding of Importin β. (**e**) and (**f**) HeLa Flp-in T-Rex cells in anaphase expressing siRNA-

resistant EGFP-tagged RepoMan-WT or RepoMan-3A, with endogenous RepoMan knocked down before fixation and immunostaining. (**g**) Recombinant His-tagged RepoMan-(1-135) was phosphorylated by Cdk2 *in vitro.* Cdk-phosphorylated RepoMan-(1-135) was incubated for 30 min at 30 °C with 100 nM PP1 purified from rabbit skeletal muscle. EDTA was added as a general kinase inhibitor to arrest phosphorylation before (lane 3) or after (lane 4) incubation with Cdk2. The dephosphorylation of RepoMan-(1-135) was followed with phospho-SP/TP antibody.

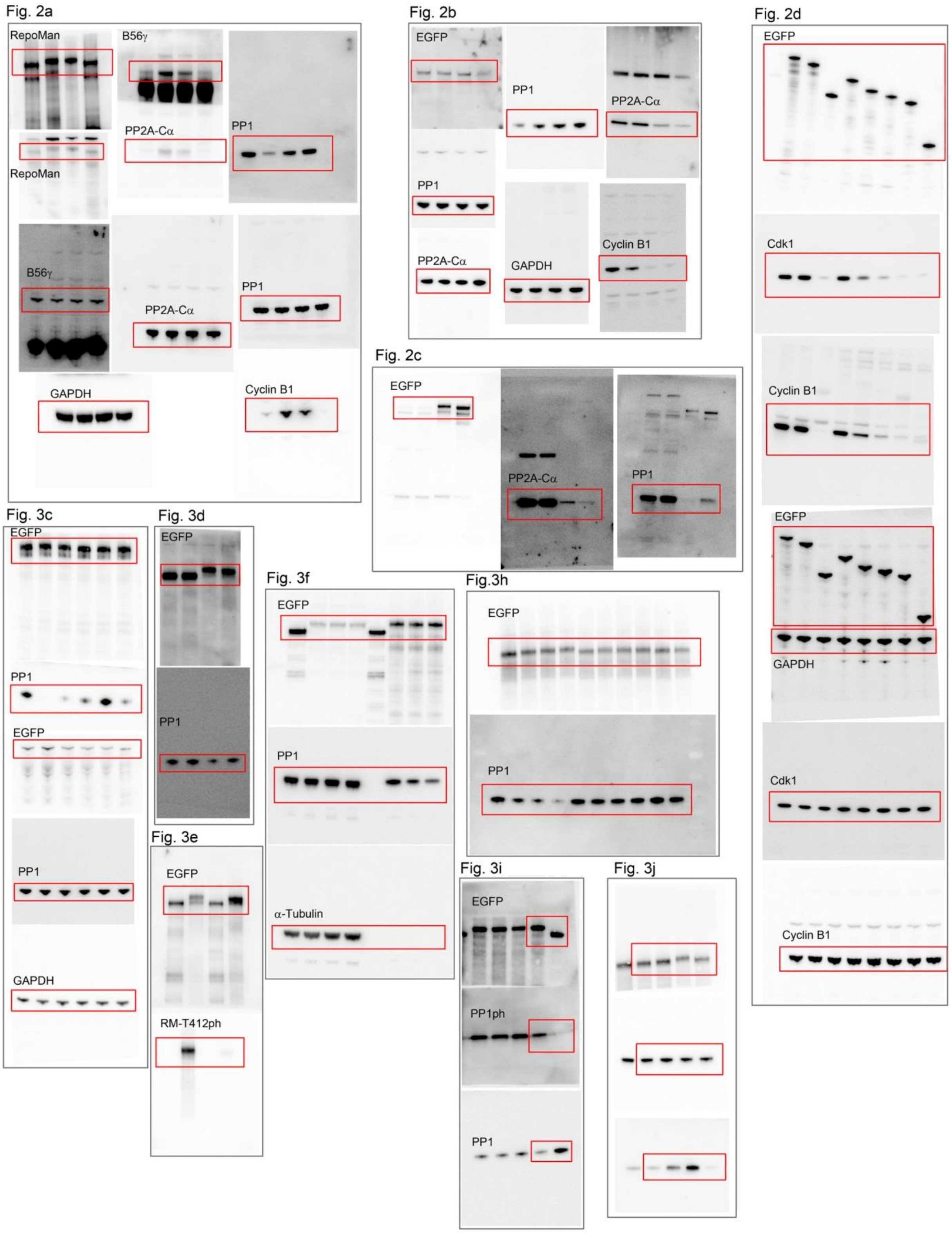


**Supplementary Figure 7. Uncropped images of immunoblots.** Red boxes show approximate image used for presentation.

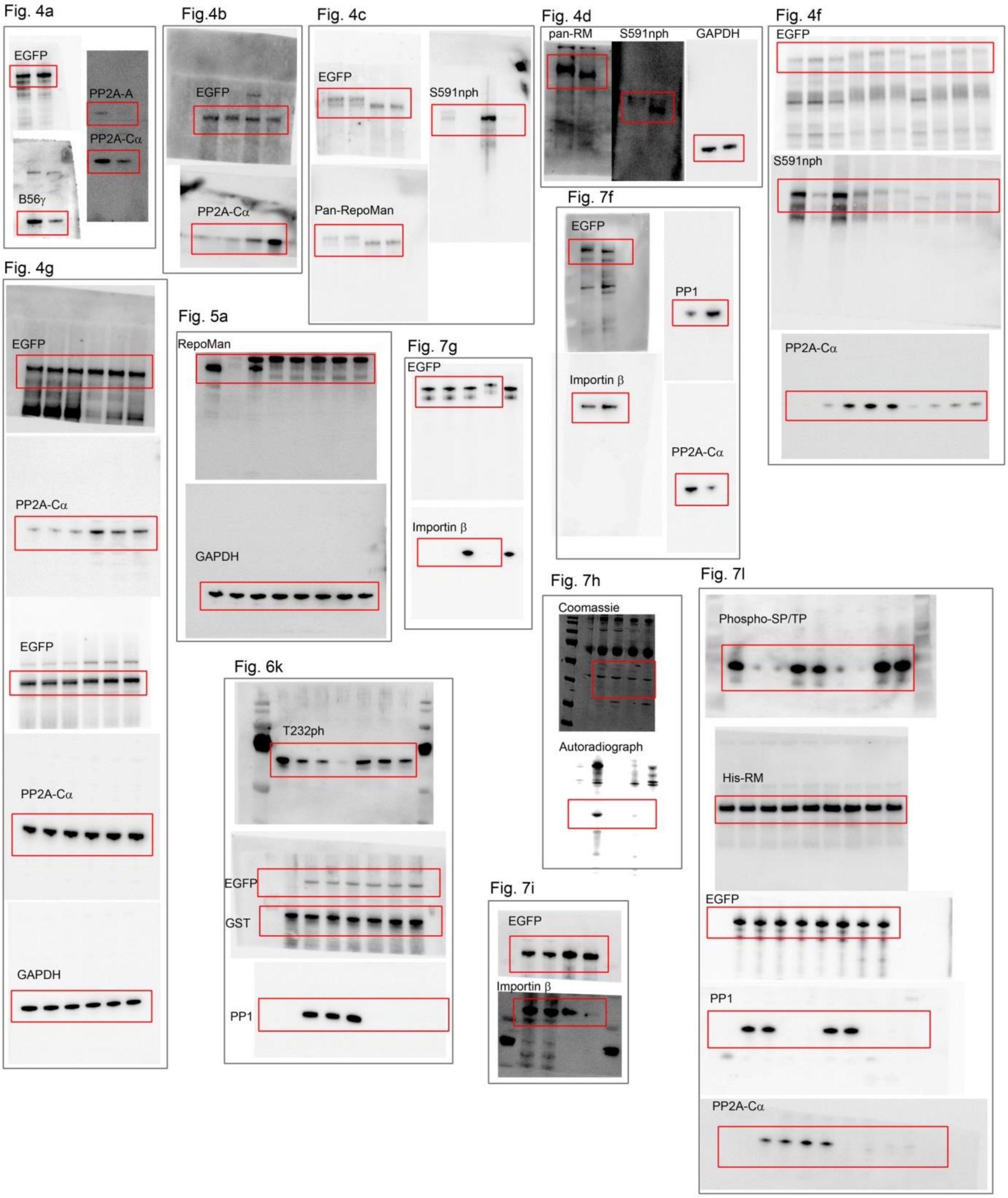


**Supplementary Figure 7. Continued.**
